# Supplementary material for: Localization of Multiple Jellyfish Toxins Shows Specificity for Functionally Distinct Polyps and Nematocyst Types in a Colonial Hydrozoan
Source: Toxins (Basel). 2023 Feb 13;15(2):149. doi: 10.3390/toxins15020149 (PMC9959030; doi:10.3390/toxins15020149)
Supplement: Supplementary file 1 [file toxins-15-00149-s001.zip › toxins-2169702-SI.pdf]

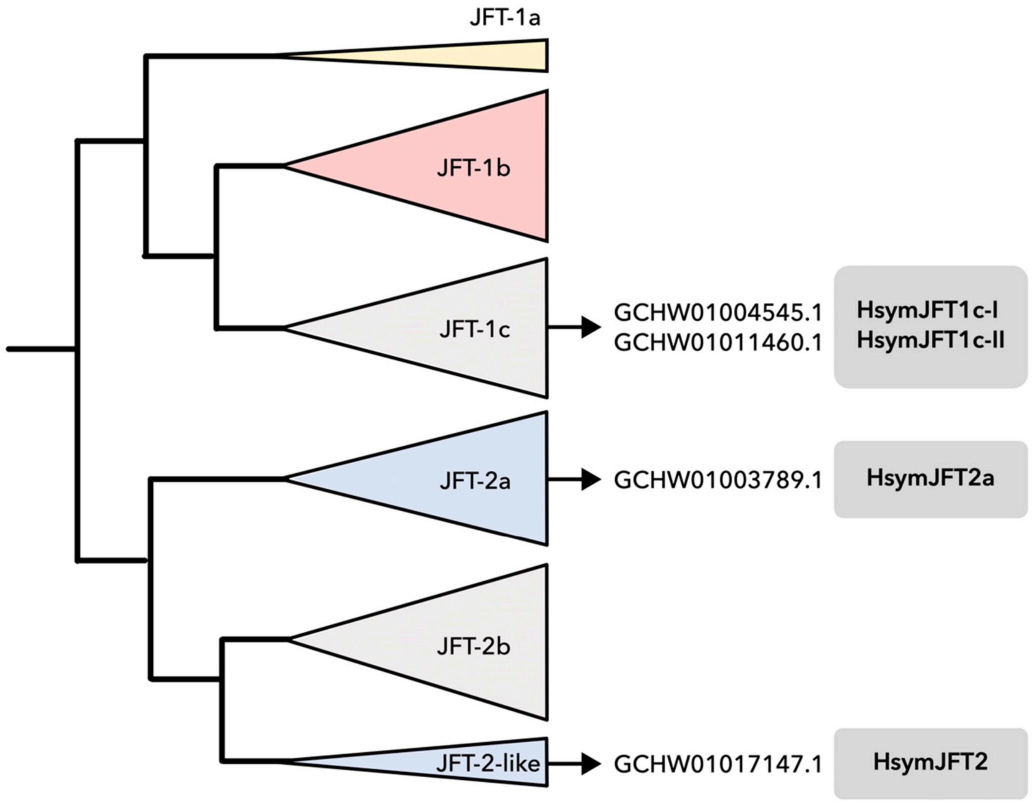

Supplementary Figure S1: Summary figure of JFT tree from Klompen et al. 2021 [32] with labeled *Hydractinia* JFTs from this work.

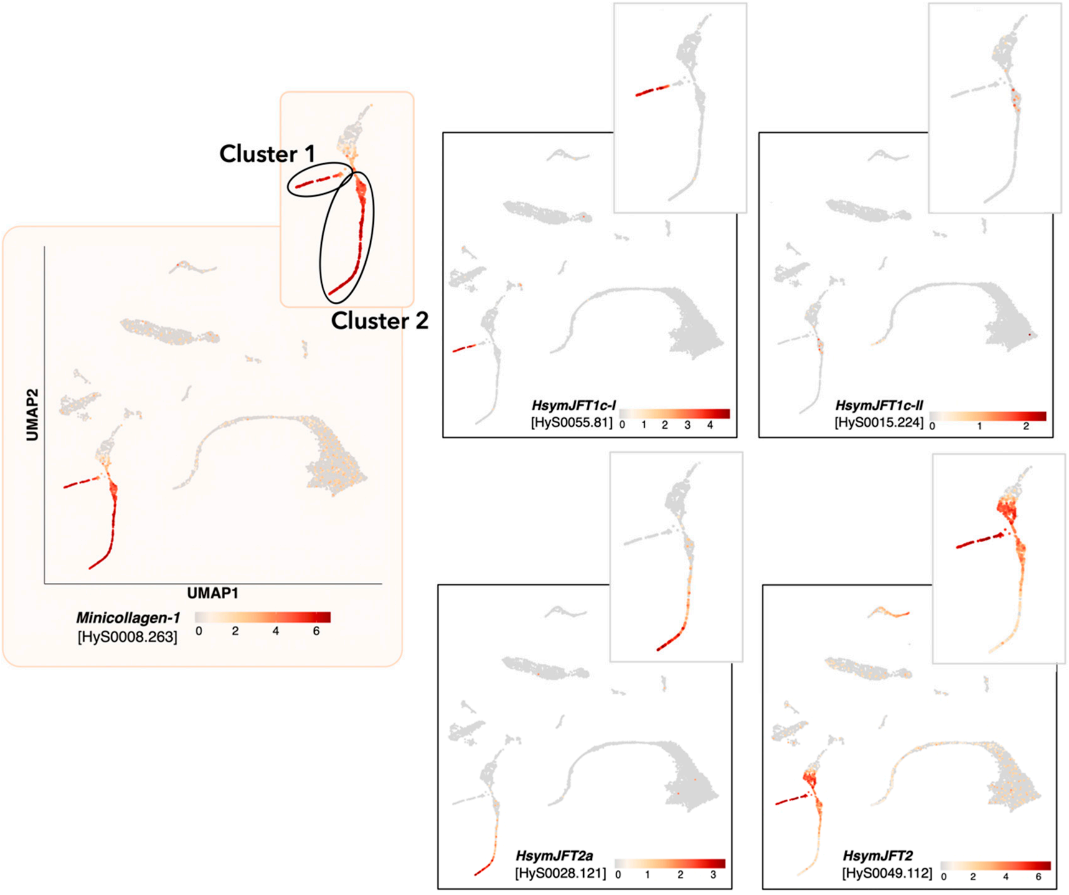

Supplementary Figure S2: JFT expression observed in *Hydractinia* gastrozooid single-cell dataset. Data accessible through the NIH *Hydractinia* portal. The expression levels for *minicollagen-1* (HyS0008.263) is shown as a proxy for cell clusters that correspond to the nematocyst lineages of cells, and Clusters 1 and 2 referenced in the main text are labeled in inset.

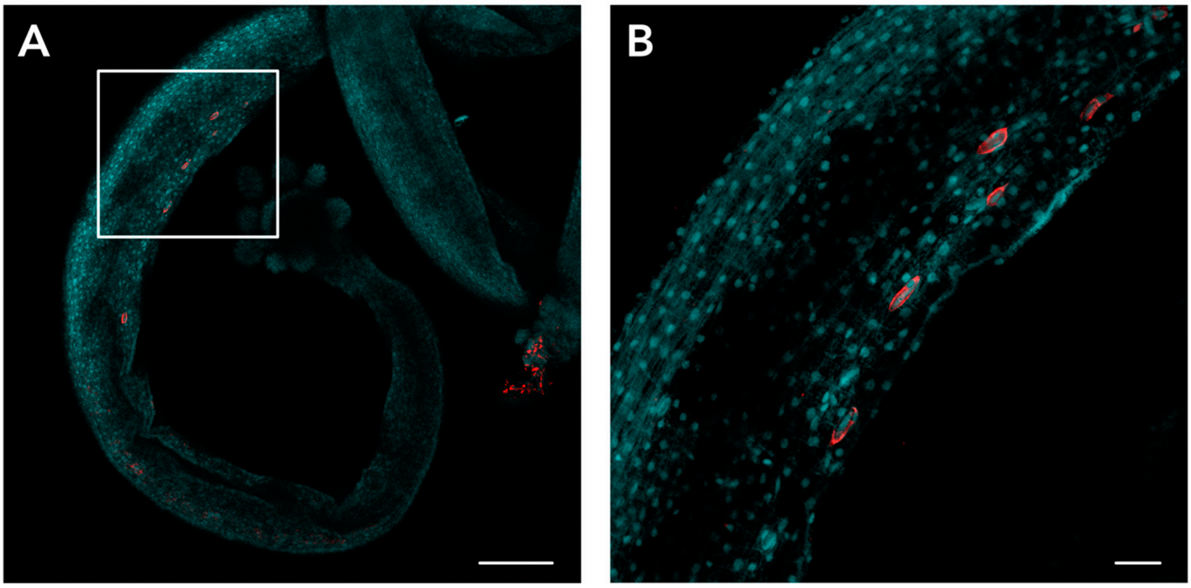

Supplementary Figure S3: HsymJFT1c-II-stained dactylozooids where subset of capsules is stained with DAPI. A) Full polyp tissue overview, where B) shows close-up image of nematocytes in body column (white box in A) that are HsymJFT1c-II-positive and capsules display DAPI signal. Cyan = DAPI, red = HsymJFT1c-II . Scale bar: 100 µm (A), 20 µm (B).

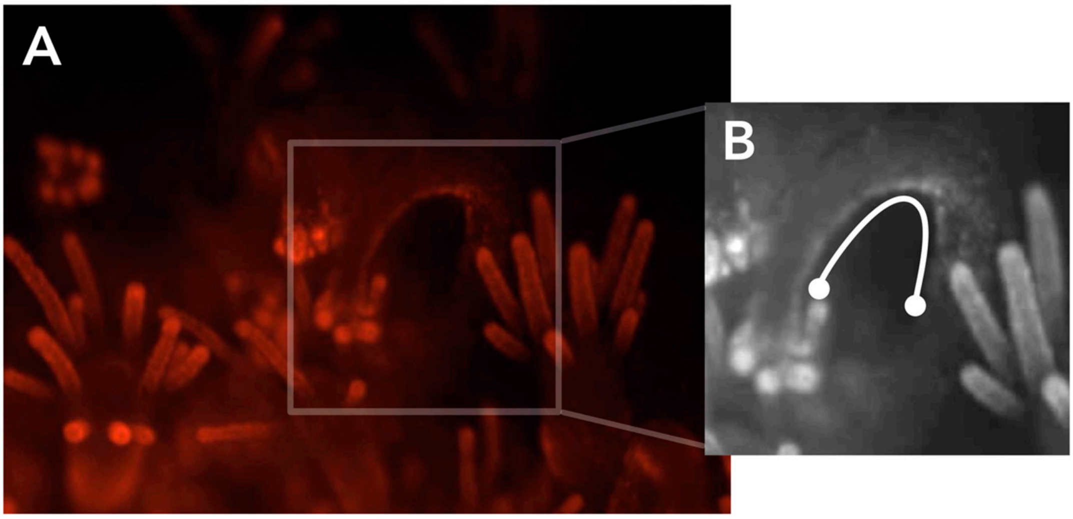

Supplementary Figure S4: Dactylozooids induced in *Ncol-1::mScarlet* colony. A) Tentacles of dactylozooids display relatively high mScarlet fluorescence signal comparable to high signal in gastrozooid body column and tentacles and in contrast to relatively low signal in gonozooid tentacles. mScarlet-positive cells on the internal of the spiral of the body column correspond to recently developing nematocysts migrating through body column along the concave region of spiral to the capitate tentacles. B) Black-and-white image of focal dactylozooid in white box of A, where white line indicates signal along the internal spiral of the body column.

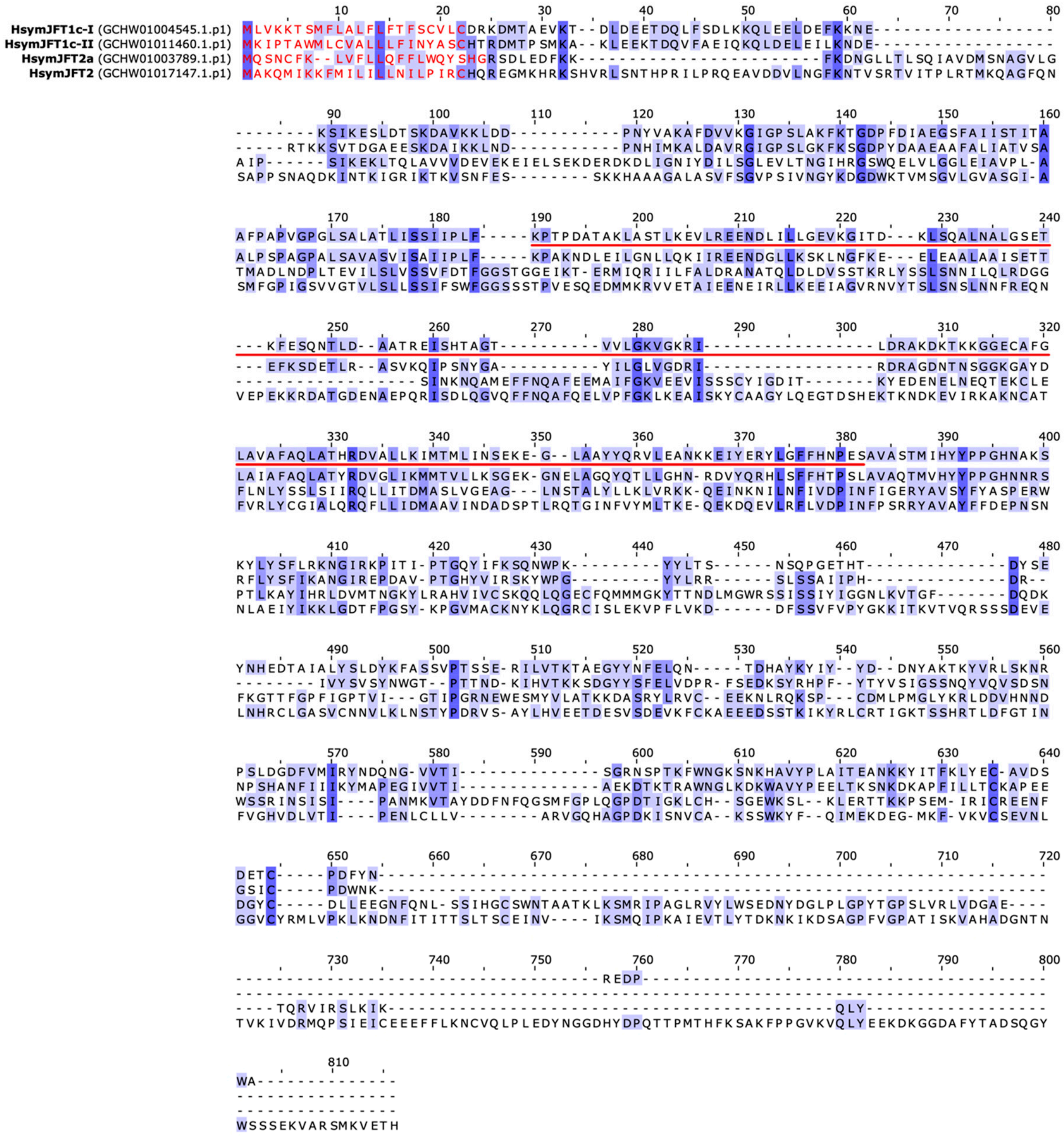

Supplementary Figure S5: Protein alignment of *Hydractinia* JFTs studied in this work. Sequences are derived from mixed tissue transcriptomic data in Klompen et al. [26] (but GCHW01003789.1.p1 = GCHW01005305.1.p1) and Sanders et al. [41]. Alignment constructed using G-INS-I algorithm in MAFFT (v7.487) [47]. Red lettering indicates signal peptides identified using SignalP (v6) [48] via the DTU Health Tech server (https://services.healthtech.dtu.dk/service.php?SignalP, assessed on 28 January 2023). The purple shading represents similarity within the alignment, such that the darker the purple background the greater the similarity between all four sequences. The red underlined fragment in HsymJFT1c-I (K120-S274) represents the antigen fragment synthesized for antibody production by ABClonal. For HsymJFT1c-II antibody, the entire protein sequence after the predicted signal peptide was synthesized by GenScript. Both antigenic regions were selected based on each company's internal quality criteria.

No signal detected with
HsymJFT1c-II staining of gastrozooid

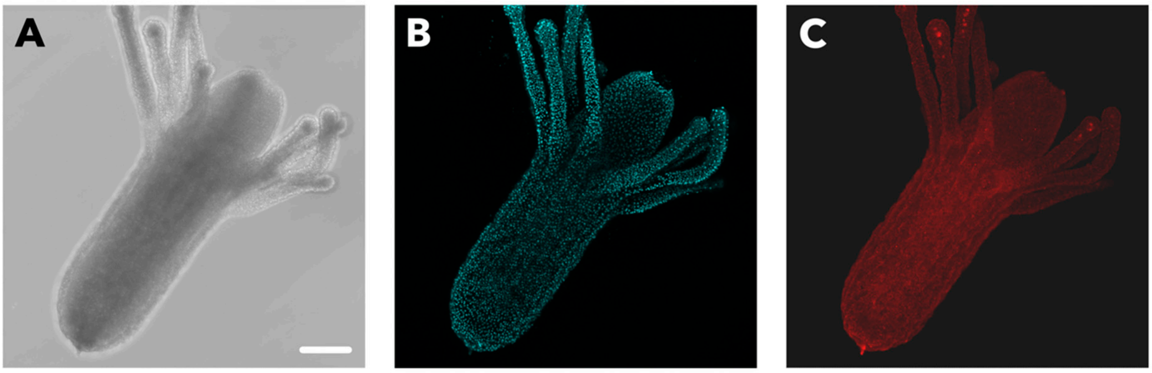

Supplementary Figure S6: Negative HsymJFT1c-II antibody staining for gastrozooid polyp tissue. A) Brightfield image of representative gastrozooid. B) DAPI-only and C) HsymJFT1c-II-only staining. Scale bar: 100 μm.

Supplementary Table S1: ISH primers designed for probe synthesis in this work.

Name	Sequence	Length (bp)
HsymJFT1c-I-ISH-F	5′- CAACGATAACTGCTGCATTCC -3′	428
HsymJFT1c-I-ISH-R	3′- CTTTAGCAACGCAACATCACG -5′	
HsymJFT2a-ISH-F	5′- GTTGTGTCTCTGCTCCATCG -3′	537
HsymJFT2a-ISH-R	3′- GTCGCTATTTGAGAGTATGCG -5′	
HsymJFT2-ISH-F	5′- GCTATCCGCCGTATAAAATGC -3′	424
HsymJFT2-ISH-R	3′- CTACCGCATGCTTGTACC -5′	
